# Analyzing Health Care Professionals’ Resilience and Emotional Responses to COVID-19 via Twitter: Retrospective Cohort and Matched Comparison Group Study

**DOI:** 10.2196/72521

**Published:** 2025-09-03

**Authors:** Noa Tal, Idan-Chaim Cohen, Aviad Elyashar, Nir Grinberg, Rami Puzis, Odeya Cohen

**Affiliations:** 1 Department of Software and Information Systems Engineering, Faculty of Engineering Sciences, Ben-Gurion University of the Negev, Beer Sheva, Israel; 2 Department of Nursing, Recanati School of Community Health Professions, Faculty of Health Sciences, Ben-Gurion University of the Negev, David Ben-Gurion Blvd 1, Beer Sheva, 8410501, Israel, 972 086477681; 3 Department of Computer Science, Shamoon College of Engineering, Beer Sheva, Israel

**Keywords:** COVID-19, emotion analysis, health care professionals, machine learning, resilience, social media, Twitter

## Abstract

**Background:**

The functioning of health care systems in emergencies relies on health care professionals (HCPs). During the COVID-19 pandemic, HCPs faced significant emotional challenges, which affected their productivity. Revealing HCPs’ emotional responses may enable the development of effective support strategies for future crises. This study examines the emotional response of HCPs and compares it with that of non-HCPs across distinct pandemic phases.

**Objective:**

The study aimed to explore and compare the emotional response of HCPs and non-HCPs during the COVID-19 pandemic, to investigate how emotional responses are linked to the pandemic’s evolution in the United States, and to assess whether the emotions experienced by HCPs can serve as an early indicator of pandemic progression.

**Methods:**

In a retrospective cohort with a matched comparison group study, we analyzed the emotions of 1450 HCPs and 1387 non-HCPs using 8,723,404 tweets collected from January 2019 to May 2022. Using Twitter profile data and a machine learning classifier, we identified HCPs and matched non-HCPs on the basis of demographics (gender, age, and ethnicity). Emotional responses (fear, sadness, joy, disgust, surprise, and anger) were examined across 5 pandemic phases (prepandemic and 4 pandemic stages) using effect sizes. We used Spearman correlations to examine the associations between HCPs’ emotions and pandemic progression.

**Results:**

HCPs displayed greater sadness (Cohen *d*=0.23) in the early pandemic phase and greater fear (Cohen *d*=0.73‐0.10) across the 3 phases than non-HCPs. HCPs exhibited lower levels of anger (Cohen *d*=−0.19 to −0.13), surprise (Cohen *d*=−0.33 to −0.15), and disgust (Cohen *d*=−0.20 to −0.18) than non-HCPs. Joy prevalence was greater among HCPs starting in the second pandemic phase (Cohen *d*=0.13‐0.16). Most emotions returned to prepandemic levels, except sadness, which remained elevated for HCPs. Emotional trends were significantly correlated with pandemic progression (*r*=0.205‐0.480; *P*<.05).

**Conclusions:**

HCPs experienced distinct emotional responses and resilience capacities compared with non-HCPs during the COVID-19 pandemic. These findings underscore the need for targeted support strategies for future emergencies. The study suggests that HCPs’ social media discussions can be complementary tools for monitoring well-being and offer preliminary signals of emerging public health concerns.

## Introduction

Health care professionals (HCPs) are essential to the functioning of the health system. During the COVID-19 pandemic, HCPs faced unprecedented challenges. They experienced extended periods of patient morbidity, elevated health risks due to exposure to an incurable disease, extended workloads, and insufficient resources [1-4]. Personal and societal disruptions affect HCPs’ ability to serve the public [3-5]. Multiple studies have confirmed the prevalence of stress, anxiety, depression, and emotional exhaustion among HCPs [6-9].

To alleviate HCPs’ emotional burden and thus enhance community resilience [10,11], we must understand the unique emotional impact of long-term emergencies on HCPs. Therefore, we designed a retrospective cohort with a matched comparison group study to compare the emotional impact of COVID-19 pandemic on HCPs versus non-HCPs. While it is reasonable to assume that HCPs experienced greater emotional impact than the general public due to their frontline exposure, the results are controversial. A meta-analysis found that the psychological burdens on HCPs and the general public were similar [12]. Phiri et al [13] revealed minimal differences in anxiety, depression, and posttraumatic stress disorder prevalence between HCPs and the general public. However, HCPs had higher rates of suicidal thoughts or self-harm, and the public experienced lower levels of well-being. A Dutch longitudinal study reported slightly better mental health among HCPs than non-HCPs during the pandemic’s first year [14]. A study among US-based HCPs found significantly higher distress levels than among non-HCPs, along with decreased proactive coping abilities [15].

Most studies on the pandemic’s impact are cross-sectional, failing to examine the changes in the pandemic’s impact over time. Existing longitudinal studies on HCPs have focused mostly on periods of 12 months or less [16-18], often including few measurement points and experiencing high participant attrition. Phiri et al [13] highlighted the need for longitudinal research on the long-term mental health impact of COVID-19 pandemic for better infrastructure to manage further emergencies.

Social media offers a pertinent data source for addressing many of the challenges of traditional longitudinal studies based on surveys. Social media–based health research has grown significantly in recent years [19], particularly in exploring the emotional impact of the COVID-19 pandemic [20]. Research has shown that different populations have exhibited unique emotional patterns throughout the pandemic [21]. Studies focusing on targeted groups, such as HCPs, have identified mental health challenges such as anxiety, depression, and emotional fatigue [22]. The evolution of emotions such as fear and anger was linked to pandemic waves, revealing dynamic emotional responses across time [23]. Tsao et al [24] identified 5 public health themes regarding the role of social media during the COVID-19 pandemic, arguing that social media can be crucial for coping with the pandemic. However, they also found that the studies faced methodological limitations, such as nonrepresentative sample sizes and selection bias.

To address the gaps described between HCPs and the general population throughout the pandemic, we analyze the emotions of a matched sample of HCPs and non-HCPs from a high-quality Twitter Panel dataset [25,26], selecting users who tweeted consistently throughout the study period from January 2019 to May 2022. The prepandemic period enables us to control for baseline differences.

The study aims to explore and compare the emotional responses of HCPs and non-HCPs during the COVID-19 pandemic, to investigate how emotional responses are linked to the pandemic’s evolution in the United States, and to assess whether the emotions experienced by HCPs can serve as an early indicator of pandemic progression. We hypothesized that HCPs would exhibit distinct emotional profiles compared with non-HCPs, with stronger correlations to pandemic metrics due to their frontline exposure and professional responsibilities.

## Methods

### Study Population

#### Dataset and Study Population Classification

We used the Twitter Panel dataset [25,26] comprising more than 1.5 million Twitter accounts associated with US public voter registration data. We trained a binary deep learning classifier (implemented using the Simple Transformers [27] Python library and Facebook’s RoBERTa model [28]) to distinguish between HCP and non-HCP Twitter accounts based on their full names and descriptions. To train the classifier, we used 1372 Twitter accounts from the dataset collected by Elyashar et al [23], which were equally divided between manually labeled HCP accounts (physicians, nurses, and mental health professionals) and non-HCP accounts (not labeled as HCP under manual definitions). These labels were derived by Elyashar et al [23] using an active learning procedure involving 20 iterations of manual inspection. In each iteration, accounts about which the model was least certain were selected for labeling. Each selected account was reviewed by 2 annotators (AE and ICC), with conflicts resolved by a third reviewer and external validation via LinkedIn when necessary. Our classifier was trained and validated using 10-fold cross-validation, achieving an accuracy of 0.827, *F*_1_-score of 0.826, precision of 0.834, and recall of 0.827.

#### Inclusion Criterion

Threshold selection was based on an iterative process, involving manual review of more than 800 accounts sampled at .01 probability intervals across the prediction range. This comprehensive sampling revealed that accounts with probabilities ≥.95 consistently exhibited high classification precision. Therefore, a threshold of .95 was selected as the HCP inclusion criterion. We applied the trained classifier with the chosen threshold to the Twitter Panel dataset, selecting 10,062 HCP accounts; manual validation of 100 random accounts confirmed that 97 were genuine HCP accounts. Similarly, for non-HCPs, we chose a likelihood threshold of 0.6 and preselected approximately 1.5 million accounts. We included user accounts that tweeted at least once a month in more than 90% (37/41 months) of the months between January 2019 and May 2022.

#### Matched Comparison Group Inclusion Criterion

We created a non-HCP sample that closely matched the HCP sample regarding gender, age, and ethnicity. Using the KDTree [29] data structure, we matched HCP accounts to similar non-HCP accounts. Statistical tests, including *t* tests and chi-square tests, confirmed the demographic similarity (Table S1 in Multimedia Appendix 1).

### Comparative Emotion Analysis

#### Extracting Emotions

We analyzed emotions using the pysentimiento [30]. Python package for evaluating Ekman’s (1995) 6 basic emotions: joy, sadness, anger, surprise, disgust, and fear [31]. For each account, we aggregated the emotions expressed in tweets during consecutive periods. Following Agarwal et al [22], we defined 5 phases during the study period: phase 1 (until February 28, 2020), phase 2 (March 1 to October 31, 2020), phase 3 (November 1, 2020, to April 30, 2021), phase 4 (May 1 to December 31, 2021), and phase 5 (January 1 to May 31, 2022). Emotion macro averages and corresponding 95% CIs were computed, ensuring that each account contributed equally regardless of the number of posts. Statistical analyses included *t* tests, Mann-Whitney *U* tests, and Wilcoxon tests for comparisons, with Bonferroni correction for multiple tests. Effect sizes across phases and subpopulations were measured using the Cohen *d*, which was based on the average of user-level emotion scores.

#### Association Between Emotions and Pandemic Progression

We used US-specific data [32] and extracted the number of new cases, hospitalizations, and deaths from July 14, 2020, according to data availability. We used the SciPy package [33] to assess weekly correlations. The analysis included examining correlations with an 8-week lag and lead between pandemic metrics and emotional responses. Only emotions with significant correlations are presented.

#### Explaining Emotions

To explain emotional dynamics, we identified the top 10% of emotionally expressive tweets and extracted the 3 hashtags most frequently used for each 3-month interval. We manually grouped the hashtags into topic categories to identify factors associated with the emotions expressed during the study period. Hashtag categorization was carried out by 2 researchers (NT and ICC) who independently reviewed the most common hashtags linked to each emotion. Themes were derived inductively, with each researcher grouping hashtags based on their semantic and contextual meaning. The researchers then compared and discussed their groupings to reach consensus. The final hashtag-to-topic mapping is available in Table S4 in Multimedia Appendix 1. We focused on hashtags related to joy, sadness, anger, and fear. In addition, we generated word clouds for the peak months (February, March, and April 2020) after text preprocessing.

### Ethical Considerations

The collection, storage, and analysis methods for the Twitter panel were approved by the institutional review board (IRB) of Northeastern University (approval number 17-12-13). Additional ethical approval, specific to this study, was obtained from the IRB of Ben-Gurion University of the Negev (approval number 1879‐1). This study analyzed publicly available data in aggregate form. No direct informed consent was obtained from users; rather, ethical safeguards were implemented in accordance with IRB guidance. To ensure confidentiality, except for the initial construction of matching groups, which was necessary to identify the HCP and non-HCP cohorts, all subsequent analysis was conducted on aggregated, deidentified data; no member of the research team examined individual accounts.

## Results

### Sociodemographics

This study included 1450 HCP accounts and 1387 non-HCP accounts, which posted 8,723,404 tweets. Approximately 1399 participants indicated their gender as female (49.33%), with a mean age of 44.11 (SD 13.19) years. No significant differences in demographic characteristics were found between the 2 groups. HCPs posted fewer tweets than non-HCPs. Table 1 describes the main demographic details (Table S1 and Figure S1 in Multimedia Appendix 1).

**Table 1.** Health care practitioners and non–health care practitioners demographics^a^.

Variable	HCPs^b^ (n=1450)	Non-HCPs (n=1387)	*P* value
Total tweets (millions), n	3.3	5.3	N/A^c^
Age average (years), mean (SD)	44.0 (13.4)	44.2 (13.0)	.681
Ethnicity (Caucasians), %	85.8	84.9	.798
Gender, %	.668
Men	45.57	48.45
Women	50.76	47.80
Unknown	3.68	3.75

a After matching, the 2 samples were not significantly different along these variables.

b HCPs: health care professionals.

c N/A: not applicable.

### Emotions During the Pandemic: Trends and Comparison

#### Comparison Between HCPs and Non-HCPs

**Figure 1.**
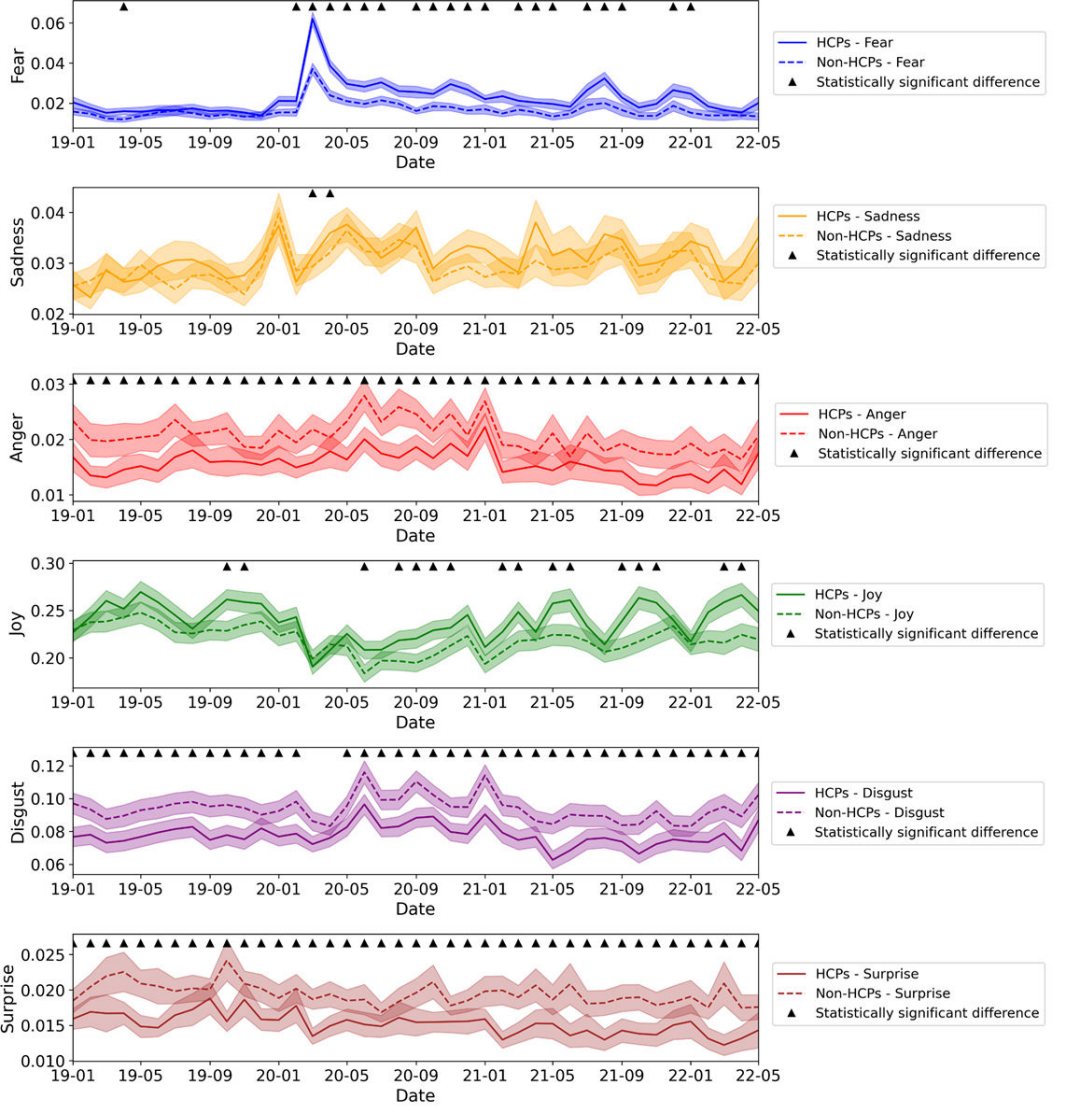
Comparison of the monthly average emotion scores of health care professionals and non–health care professionals with 95% CIs. HCPs: health care professionals; non-HCPs: non–health care professionals.

**Figure 2.**
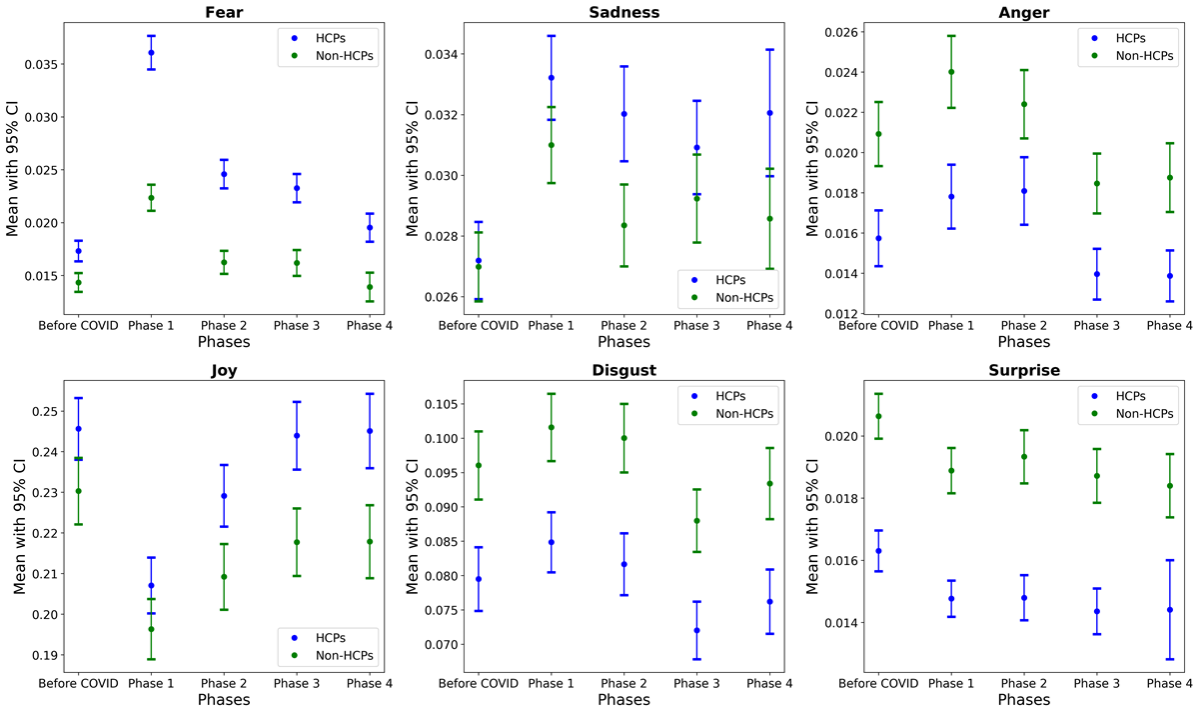
Averages of 6 emotional responses for health care professionals and non–health care professionals across study phases. Average emotion scores based on pandemic phases as described by Agarwal et al [22]. HCPs: health care professionals; non-HCPs: non–health care professionals.

Figures1  2 depict average emotion scores for the study populations during the study timeline. Figure 1 shows monthly averages, with triangles indicating significant differences between HCPs and non-HCPs, while Figure 2 displays the average emotions aggregated according to the 5 study phases. Figure 1 shows that HCPs’ fear scores rise sharply at the onset of the pandemic and remain consistently higher than those of non-HCPs. In contrast, their anger, surprise, and disgust scores remained consistently lower across most of the period. Notably, CI values for the groups showed minimal overlap, especially in periods with significant group differences marked by triangles, most prominently in fear, anger, and joy. Figure 2 reinforces these trends by phase, with clearer group separations in fear, anger, and joy, indicated by largely nonoverlapping error bars.

Quantitatively, HCPs exhibited lower levels of anger (Cohen *d*=−0.19 to −0.13), surprise (Cohen *d*=−0.33 to −0.15), and disgust (Cohen *d*=−0.20 to −0.18). In addition, HCPs expressed significantly higher fear levels than non-HCPs throughout the pandemic phases (Cohen *d*=0.22‐0.5) but not before the pandemic. HCPs exhibited higher levels of joy (Cohen *d*=0.1‐0.16) from the second phase of the pandemic than non-HCPs did (Figure 1 and Table S2 in Multimedia Appendix 1). Regarding emotional recovery, the recovery curve for HCPs was steeper than that of non-HCPs in terms of joy and fear (Figure 2).

#### Emotional Patterns of Both Populations Across Study Periods

HCPs expressed higher levels of fear and sadness and lower levels of joy compared with the prepandemic period. This trend persisted through the first 3 phases of the pandemic, with fear remaining particularly high during the initial stages (Cohen *d*=0.73, 95% CI 0.656‐0.806 in phase 1; Cohen *d*=0.32, 95% CI 0.245‐0.392 in phase 2; and Cohen *d*=0.26, 95% CI 0.188‐0.334 in phase 3). Examining the magnitude of change between HCPs and non-HCPs, fear demonstrated a greater change during the first 3 periods of the pandemic, with no overlap in the 95% CIs of the Cohen *d* effect sizes. In the last phase of the pandemic, all emotions returned to prepandemic levels except for sadness, which remained slightly higher (Cohen *d*=0.145, 95% CI 0.072‐0.218). Among non-HCPs, compared with the prepandemic period, joy decreased (Cohen *d*=−0.23, 95% CI −0.305 to −0.155), while fear (Cohen *d*=0.39, 95% CI 0.319‐0.470) and sadness (Cohen *d*=0.18, 95% CI 0.103‐0.253) increased during the first pandemic phase, with no additional significant effect sizes in later phases (Table S3 in Multimedia Appendix 1).

#### Correlation Between Emotions and Pandemic Progression

**Figure 3.**
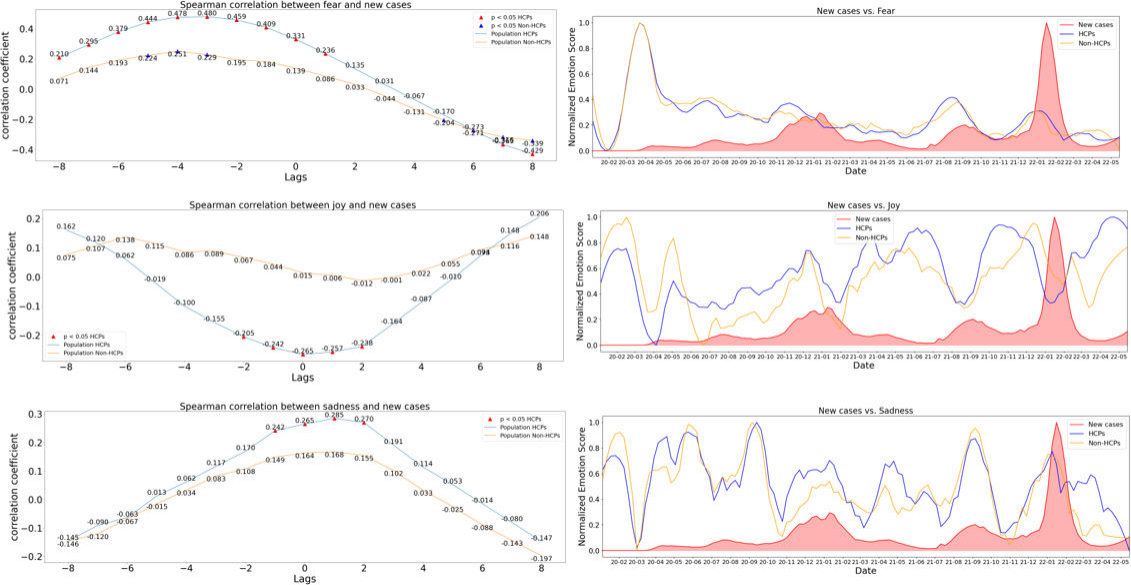
Correlations between fear, joy, sadness, and pandemic progression. Left: Spearman correlation between emotions and new cases with lags of −8 to 8 weeks; significant correlations (*P*<.05) are highlighted with triangles. Right: Smoothed and normalized emotion scores compared with new cases over time. HCPs: health care professionals; non-HCPs: non–health care professionals.

We examined the correlation between population emotions and COVID-19 progression by analyzing how weekly emotion scores related to new case counts at different time lags. In the visual analysis (Figure 3), the left panels show the correlation coefficients for each emotion over a range of time lags (with triangles indicating significant associations), while the right panels align the smoothed and normalized emotion scores with pandemic waves, making it possible to observe how peaks in emotions coincide with fluctuations in case numbers.

This analysis shows that HCPs’ fear, sadness, and joy were more strongly associated with new COVID-19 pandemic cases than they were among non-HCPs. No significant correlation with pandemic measurements was found for other emotions. A significant correlation between HCPs’ sadness and new cases emerged (ρ=0.242; *P*=.016), with sadness increasing 1 week after a respective increase in new cases. HCPs’ fear levels were most strongly correlated with new cases of COVID-19 disease reported 3 weeks prior (ρ=0.480; *P*<.001), and a significant decrease in HCPs’ joy levels was observed with an increase in new cases without a lag (ρ=−0.205; *P*=.044). Figure 3 summarizes the correlations between emotional trends and pandemic progression across an 8-week lag, alongside the timeline of emotions during the pandemic.

#### Explaining Emotion Based on Hashtag Usage Analysis

**Figure 4.**
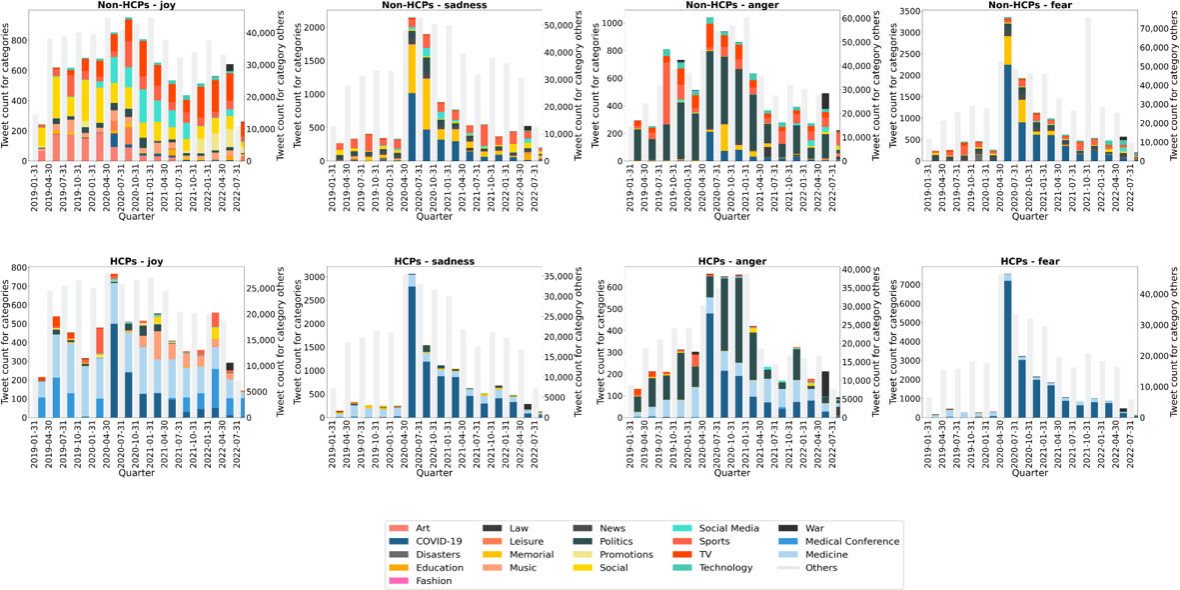
Analysis of hashtags related to the 4 primary emotions: joy, sadness, anger, and fear. The top row of graphs presents data for non–health care professionals, and the bottom row presents data for health care professionals. The frequency of specific categories is indicated on the y-axis, while the right y-axis is the count of tweets belonging to the “others” category. The quarters analyzed are indicated on the x-axis. Categories, indicated by color, represent hashtags extracted from the top 10% of emotionally expressive tweets. HCPs: health care professionals.

Figure 4 depicts the prevalence of different hashtag types for the 2 study populations. Additional information about hashtag classification is given in Table S4 in Multimedia Appendix 1.

##### Joy

Among categorized tweets in the upper decile of joy, 81.84% (5289/6463) of HCPs’ tweets focused on professional topics, whereas non-HCPs’ tweets conveyed joy across a broader range of topics.

##### Sadness

Since January 2020, among categorized tweets in the upper decile of sadness, COVID-19 pandemic was the predominant source of sadness (5093/5974, 85.25%) among HCPs. In contrast, from the end of July 2021, sadness among non-HCPs began focusing on a broader range of topics.

##### Fear

Tweets with high fear levels that included topic hashtags primarily focused on COVID-19 pandemic for HCPs and non-HCPs. While popular non–COVID-19 hashtags within fearful HCP tweets were related to medicine (1060/20,737, 5.11%), non-HCPs showed a greater diversity of fearful content.

##### Anger

In the upper decile of anger tweets, those from non-HCPs predominantly addressed political content (5510/8104, 67.99%), while those from HCPs focused on COVID-19 pandemic (1602/4763, 33.63%). After April 2020, HCPs continued to concentrate on the COVID-19 pandemic, with a gradual shift toward categories related to politics (1818/4763, 38.16%). Word clouds of peak-month tweets were generated for each emotion, highlighting similarities and differences between HCPs and non-HCPs (List S1 in Multimedia Appendix 1).

Word clouds of peak-month tweets were generated for each emotion, highlighting similarities and differences between HCPs and non-HCPs (List S1 in Multimedia Appendix 1).

## Discussion

### Principal Findings

We analyzed emotions expressed by US HCPs and non-HCPs from January 2019 to May 2022. Given the increasing frequency of natural disasters, events such as the COVID-19 pandemic are likely to become more common, making it crucial to gain insights into the lessons learned for future prolonged emergencies [34]. Although this study did not measure mental health outcomes, the observed emotional patterns serve as meaningful proxies for participants’ mental health [35,36].

Our analysis revealed significant emotional turmoil in both groups during the COVID-19 pandemic. HCPs experienced notably higher fear levels, which is consistent with the findings by García-Fernández et al [37], who reported elevated fear in 58.5% of HCPs. Additionally, our study found that HCPs’ sadness levels remained above pre-COVID-19 levels during the final phase of the pandemic (Figure 2). Thus, similar to the patterns observed in posttraumatic stress disorder symptoms, after exposure to stress, negative emotions endure more strongly than before the stress-inducing event [38]. Given HCPs’ critical role during emergencies, systemic support is essential to maintain their capacity.

HCPs expressed higher levels of joy than non-HCPs did from the second COVID-19 outbreak phase onward. Muthuri et al [39] noted that happiness among HCPs reflects the meaning and value they find in their work. During the pandemic, this could be associated with a sense of purpose and fulfillment, as well as the appreciation they received from patients and families. This aligns with findings by McHugh et al [40], who highlighted that HCPs’ well-being during the pandemic was significantly enhanced by their sense of identity and purpose at work.

We found that HCPs exhibited lower levels of anger, surprise, and disgust than non-HCPs. There is limited literature on this issue. However, our results contrast with those of García-Fernández et al [37], who reported higher levels of anger among HCPs. Our findings could be related to the emotional regulation required for good practice in health care delivery [41]. Another reason may be the professional knowledge HCPs possess regarding stress-inducing events, moderating these feelings [42].

Personal resilience is the maintenance of quick recovery during or after stressor exposure [43]. Resilience reflects the adaptive capacity for change and is perceived as a core component of emergency response. During the COVID-19 pandemic, HCPs exhibited emotional recovery despite experiencing more distinct changes in emotional responses relative to non-HCPs. High levels of joy, along with low levels of anger, disgust, and surprise, indicate resilience among HCPs. Resilient HCPs ensure the continuity of health care services during emergencies and contribute to community resilience since their role broadens beyond the mere provision of medical treatment [10,11].

Our findings show that HCPs’ tweets with high emotion scores were often associated with professional topics across all emotions, which validates that emotional expression differences are work-related. The study supports the claim that HCPs’ professional identity extends beyond their workplace, influencing their social media interactions [44]. Fear in both HCPs and non-HCPs was associated with the progression of the pandemic. However, HCPs expressed additional concerns associated with their work environment, such as protective equipment, and the challenge of balancing professional commitment and personal safety. Our hashtag analysis supports this assumption, revealing that HCPs’ joyful tweets during the early stages of the pandemic focused primarily on professional topics, often using work-related terms such as “proud work.”

### Comparison With Previous Work

Comparing our findings with previous social media studies reveals both shared and distinct emotional response patterns. Consistent with our results, Saha et al [45] and Ashokkumar and Pennebaker [46] identified elevated levels of anxiety and sadness among non-HCPs early in the pandemic, while Guntuku et al [47] observed a similar trend in expressions of joy among this group. Ashokkumar and Pennebaker [46] also reported decreased anger during the pandemic. In contrast, our findings indicate nonsignificant changes in anger among non-HCPs across all phases, with a slight increase in the early phases, followed by a gradual decrease. This difference may be due to variations in the study populations and platforms.

Our HCPs’ findings align with those of the Twitter analysis by Agarwal et al [22], which reported initial emotional distress that gradually stabilized. Both studies observed emotional fluctuations: joy declined in phase 1, partially rebounded in phase 2, and stabilized by phases 3 and 4. Fear (in our study) and anxiety (in the study by Agarwal et al [22]) surged in phases 1 and 2 before a gradual decrease. Sadness in our data parallels the findings by Agarwal et al [22] on depression and loneliness, with an early rise followed by a partial decline, though without full recovery. Anger initially rose and then decreased, with our study showing a milder and statistically insignificant increase.

### Strengths and Limitations

This study has several strengths. The temporal scope extends from January 2019 to May 2022, capturing prepandemic, pandemic, and recovery phases. The comparative design between HCPs and non-HCPs enables the identification of occupation-specific emotional patterns. A participation-based inclusion criterion—requiring users to tweet at least once a month in more than 90% of the study months—ensured consistent user engagement and reduced noise from inactive accounts. The analysis incorporates multiple emotional dimensions rather than single affect measures and provides comprehensive emotional profiling. The large-scale social media dataset supports robust statistical analysis across pandemic phases.

Several limitations should be noted. First, the study analyzed a US sample and included only those HCPs and non-HCPs who use Twitter, excluding nonusers. Second, the nature of Twitter limits the depth of information compared with other sources, such as interviews. Third, it relies on emotions as proxies for mental health outcomes.

### Future Directions

Several studies have explored social media monitoring to detect or predict COVID-19 pandemic [41]. For example, Ockerman and Carrier [48] predicted COVID-19 case counts using Twitter images. Shen et al [49] reported that reports of symptoms and diagnoses of COVID-19 disease on social media significantly predicted daily case counts up to 14 days ahead of official statistics. Our findings suggest that analyses of HCP social media posts should yield more informative preliminary signals of emerging public health concerns than those of the general population.

Future research should examine cross-platform emotional patterns beyond Twitter to validate findings across social media environments. Integrating emotional data from social media with clinical mental health assessments could strengthen the validity for emotional proxies. The development of real-time monitoring systems for HCPs’ well-being could enable timely interventions during health emergencies.

### Conclusions

Learning from the COVID-19 pandemic is essential for preparing for future prolonged emergencies. This study found that HCPs and non-HCPs experienced significant emotional turmoil during the COVID-19 pandemic. However, the emotional impact of the pandemic was more pronounced among HCPs than non-HCPs. This study highlights the resilience and professional identity of HCPs as reflected in social media. We propose that HCPs’ social media activity may serve as an indicator of emergency progression and a complementary source for assessing their well-being.

## Supplementary material

10.2196/72521 Multimedia Appendix 1 Health care professionals’ resilience and emotional responses to COVID-19 via Twitter.

## Abbreviations

HCP: health care professional
IRB: institutional review board
