# Supplementary material for: Analyzing Health Care Professionals’ Resilience and Emotional Responses to COVID-19 via Twitter: Retrospective Cohort and Matched Comparison Group Study
Source: J Med Internet Res. 2025 Sep 3;27:e72521. doi: 10.2196/72521 (PMC12408095; doi:10.2196/72521)
Supplement: Multimedia Appendix 1 [file jmir-v27-e72521-s001.docx]

##

**Figure S1.** Pipeline.
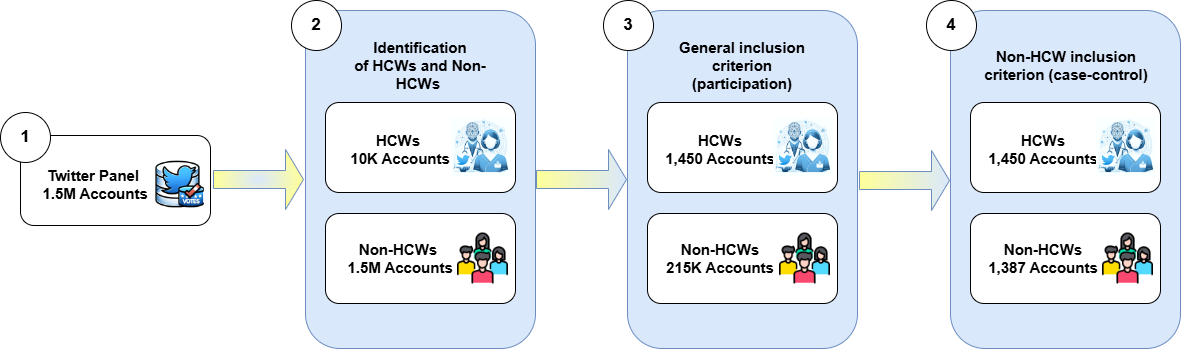


**Table S1.** HCWs and non-HCWs statistics test.

| Feature | Test | *P* value | Statistic |
| --- | --- | --- | --- |
| Age | T-test | 0.68 | 0.41 |
| Gender | Chi-square test | 0.67 | 2.37 |
| Race | Chi-square test | 0.80 | 0.06 |

**Table S2.** Statistical analysis of monthly emotional differences across populations.

**
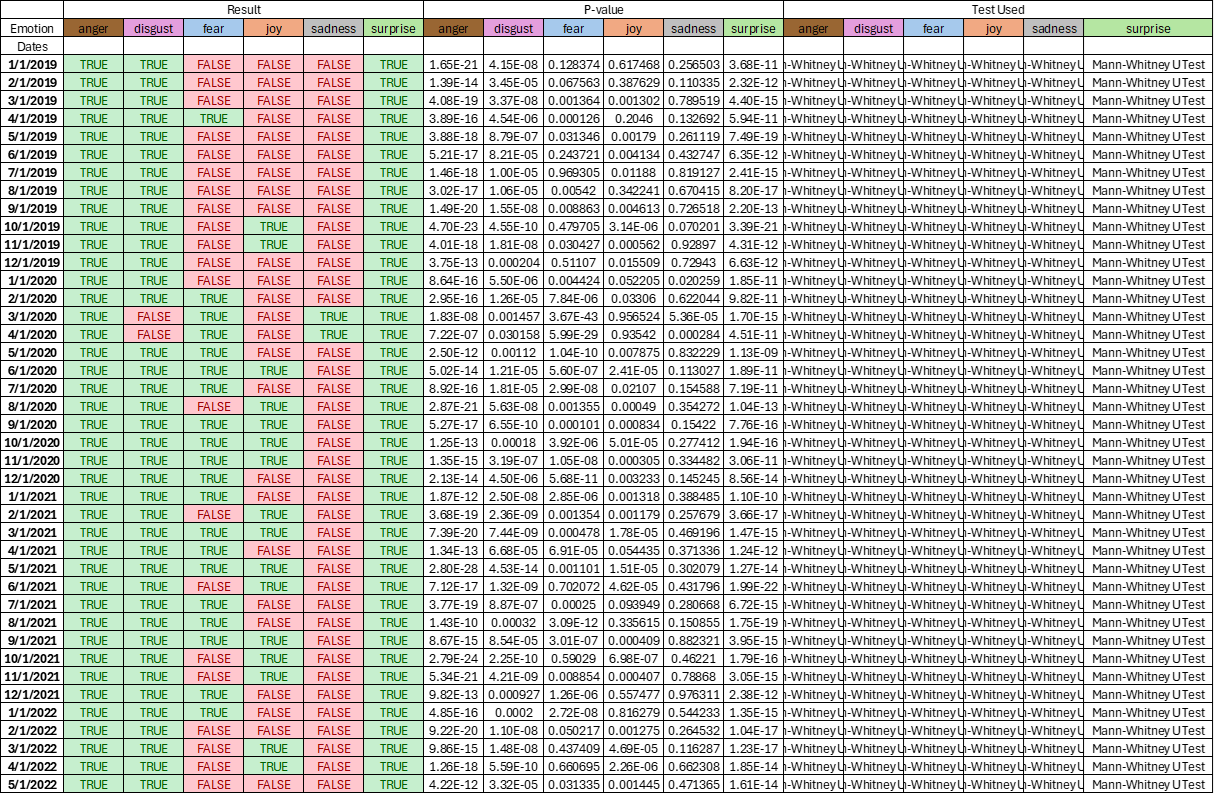
**

**Table S3.** Cohen *d* tests for within and between population comparisons.


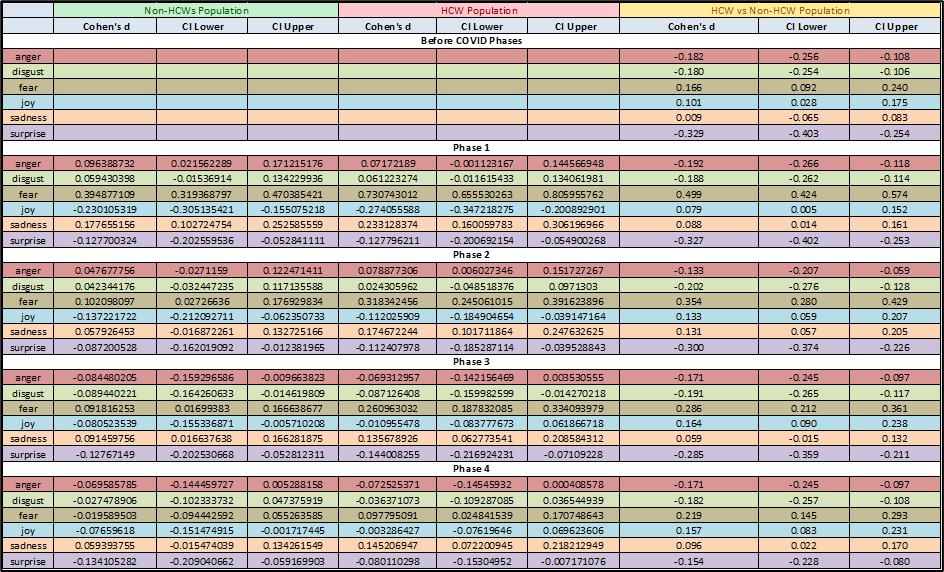


**Table S4.** Hashtags to topics.

| **Hashtags** | **Topics** |
| --- | --- |
| #RSNA19, #AGS22, #ASH21, #ASPNR2019, #ACC19, #ATS2022, #ACC22, #ASNR22, #ACR2019, #ACG2021, #ASCO21, #ANES21, #EAST2019, #APhA2019, #RSNA21 | Medical Conference |
| #Prayer, #AkashicRecordsWisdom | Religion |
| #photography,#photos | Art |
| #GetVaccinated, #MedEd, #MedTwitter, #RITPatientCare, #StopAntiVaxPropagand, #VaccinesWork, #WomenInMedicine, #cannabis, #flu, #healthcare, #hpm, #measles, #medtwitter, #mentalhealth, #monkeypox, #PedsICU | Medicine |
| #Cowboys, #GeauxTigers,#Dodgers, #GoBuckeyes,#NYR, #Padres, #WPMOYCHALLENGEGOULD, #Yankees, #getbetter, #iahsbb, #iahsbkb, #iahsbsb, #iahsfb, #iahssb, #iahswr | Sports |
| #Bitcoin, #infosec, #cybersecurity | Technology |
| #LivePD, #Gotham, #AEW, #AEWDoN, #AEWDynamite, #AEWRampage, #Horror, #MorningJoe, #VeryScaryPeople, #criticalrolespoilers, #horror, #morningjoe, #scaryhorrorstuff | TV |
| #style, #fashion | Fashion |
| #FunkoPop, #Funko, #6amcrew, #criticalrole | Leisure |
| #BlackHistoryMonth | Education |
| #FIX19, #CCC49, #FBI, #SDCC, #ScrubsNHeels22, #SteeleInsiders, #professionalism, #steeleinsiders, #thelineman,#1,'#FBI,'#Ida,#Topbuzz, #WTF,#realestate | Others |
| #coronavirus, #COVID19, #covid19, #StopTheOutbreaks, #Omicron, #COVID | COVID-19 |
| #thebandsteele, #musicvideo, #TheBandSteele, #HipHopEd | Music |
| #Sweepstakes, #giveaway, #BookBuzzr, #Poshmark, #Promo, #shopmycloset | Promotions |
| #GunViolence, #WPMOYChallenge, #EmpowermentMoment, #EmpowermentRevolution, #Foster, #NYCACC, #Pledge, #Rescue, #ShareTheLove,#TN | Social |
| #NeverForget, #HolocaustMemorialDay, #MemorialDay | Memorial |
| #Afghanistan, #AmericasGreatestMistake, #BeBest, #Biden,  #BidenHarris2020, #BidenIsADisgrace,#BlackLivesMatter,#DerangedDonald, #GunControlNow, #ImpeachTheMF, #ImpeachTrump, #MAGA, #MoscowMitch, #MuellerReport,#SOTU,#Trump,#TrumpBetrayedTheKurds,#TrumpIsALoser,#TrumpResign,#TrumpShutdown, #Bernie,#Debates2020, #DemDebate, #DementedJoe, #DerangedDonald, #GOPTraitorsToDemocracy,#IdiotInChief, #ImpeachAndRemoveTrump, #ImpeachTheMF, #ImpeachmentInquiryNow, #M4A, #MoscowMitch, #NeverBernie, #Putin, #StopTheSteal, #TrumpKnew, #TrumpResign, #TrumpShutdown, #TrumpTapes, #TrumpVirus, #Va, #lagov, #lalege, #trumpisafuckingidiot | Politics |
| #DerekChauvinTrial, #RoeVWade | Law |
| #SlavaUkraini, #Mariupol, #Ukraine, #Russia, #RussianWarCrimes | War |
| #SciComm, #TikTok, #CoreChat,#SOTD,#TopNewFollowers,#TwitterACritter,#fearporn,#twitch | Social Media |
| #BREAKING | News |
| #Dorian,#Unvalde, #NotreDame, #HurricaneDorian | Disasters |

List S1. Different hashtags’ types across sub-populations:

For joy, both mentioned "stay_safe". However, HCWs emphasized work-related terms (e.g., "proud_work","congratulation_dr"), while non-HCWs focused on general topics like "god_bless". Shared sad hashtags included "tested_positive" and "new_york", with HCWs using professional terms like "healthcare_worker" and non-HCWs expressing personal experiences ("sorry_loss"). For anger, both referenced political figures and topics like "donald_trump" and "public_health", but HCWs highlighted "health_care”, while non-HCWs used general political terms like "fake_news". Concerning the fearest hashtags, both focused on pandemic-related fears ("coronavirus_death", "tested_positive"). HCWs emphasized "protective_equipment", while non-HCWs referred to less stressful terms like "coronavirus_case".

**Figure S2.** Hashtags and word clouds.


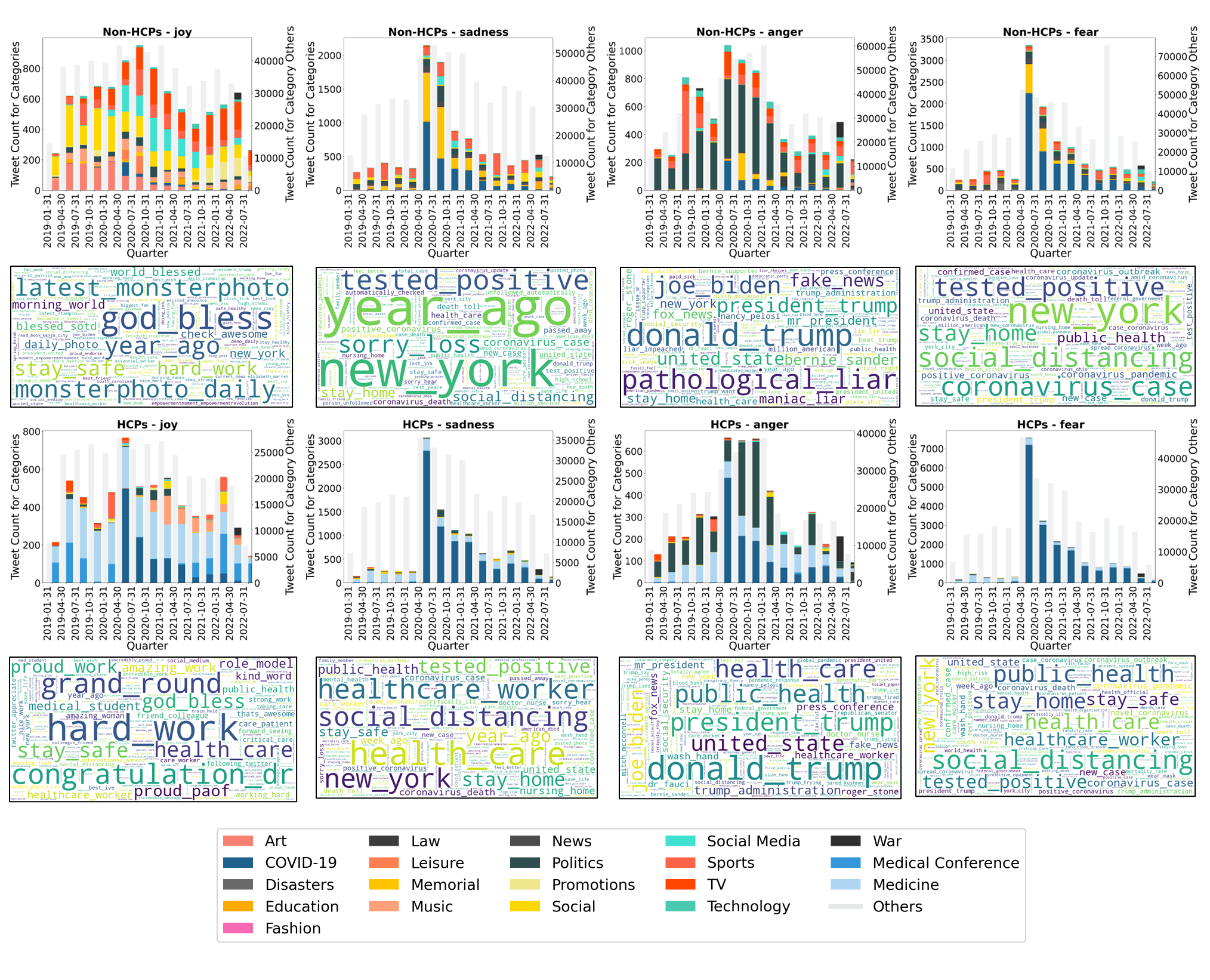


Analysis of hashtags related to the four primary emotions: joy, sadness, anger, and fear. Note. The top row of graphs presents data for non-HCP, and the bottom row presents data for HCPs. The frequency of specific categories is indicated on the y-axis, while the right y-axis is the count of tweets belonging to the ‘“others”’ category. The quarters analyzed are indicated on the x-axis. Categories, indicated by color, represent hashtags extracted from the top 10% of emotionally expressive tweets. Word clouds represent each emotion’s leading words from February, March, and April. HCPs: health care professionals; Non-HCPs: non-health care professionals.
